# Transcriptional and post-translational regulation of aquaporins in plants

**DOI:** 10.1007/s44154-026-00301-9

**Published:** 2026-04-13

**Authors:** Chao Yang, Milca Banda Medison, Yuping Li, Yi Shi, Rudoviko Galileya Medison, Huanfang Liu, Ying Wang

**Affiliations:** 1 Guangdong Provincial Key Laboratory of Applied Botany, South China Botanical Garden, Chinese Academy of Sciences, Guangzhou, 510650 China; 2 University of Chinese Academy of Sciences, Beijing, 100049 China; 3 State Key Laboratory of Plant Diversity and Specialty Crops, South China Botanical Garden, Chinese Academy of Sciences, Guangzhou, 510650 Guangdong China

**Keywords:** Aquaporins, Transcriptional regulation, Phosphorylation, Ubiquitination, Stress adaptation

## Abstract

Aquaporins (AQPs) are channel proteins belonging to the major intrinsic protein (MIP) superfamily that mediate the transport of water and other small uncharged molecules across biological membranes. They play essential roles in maintaining cellular homeostasis during plant growth and environmental adaptation. Studies on the molecular regulation of AQP expression and post-translational modification have unveiled an exquisite panorama. The expression of *AQP* genes is dynamically regulated in response to external and internal signals. Stress- and development-responsive transcription factors (TFs) from AP2/ERF, MYB, NAC, and WRKY families, govern the transcriptional reprogramming in response to drought, salinity stress and/or developmental cues. Besides, post-translational modifications (PTMs), such as residue-specific phosphorylation by kinases (e.g., CPKs, SnRK2s) and ubiquitination by E3 ligases (e.g., AtRma1, OsHIR1), precisely modulate AQP activity and/or stability. This review synthesizes current knowledge on the molecular mechanisms of transcriptional regulation and PTM-driven fine-tuning of AQPs, emphasizing their important roles in optimizing plant growth resilience under fluctuating environmental conditions.

## Introduction

Plant growth and development depend on strict water transport and regulation. In this process, aquaporins (AQPs) play vital roles. These small hydrophobic channels with a molecular mass ranging from 26 to 34 kDa usually function as tetramers (Daniels et al. 1999; Kukulski et al. 2005; Törnroth-Horsefield et al. 2005). Each protein consists of six transmembrane α-helices linked by five loops (A–E), with their N- and C-termini located in the cytoplasm. The cytosolic loop between the second and third transmembrane domains (loop B) and the extracytosolic loop between the fifth and sixth transmembrane domains (loop E) both form short hydrophobic helices that dip halfway into the membrane from opposite sides (Daniels et al. 1999; Kukulski et al. 2005; Törnroth-Horsefield et al. 2005). In addition to water, AQPs are also found to facilitate the transport of many small neutral solutes across biological membranes, including glycerol, reactive oxygen species (e.g., H₂O₂), gas signaling molecules (e.g., NO, H₂S, NH₃, CO₂), and essential metalloids like arsenic and boron (Sun et al. 2024; Maurel et al. 2015; Handa et al. 2022; Li et al. 2024). This multifunctional transport capability underpins AQPs' involvement in key physiological processes ranging from osmoregulation and nutrient partitioning to stress signaling, enabling developmental plasticity and environmental adaptation of plants (Wang et al. 2020; Tang et al. 2023).

The first plant aquaporin, Nodulin-26 (GmNOD26), was isolated from soybean (*Glycine max*) in 1987 (Fortin et al. 1987). However, their water transport activity was not evidenced until 1993, when Maurel et al. showed the functionality of an *Arabidopsis thaliana* tonoplast intrinsic protein homolog (AtTIP1) using *Xenopus laevis* oocytes (Maurel et al. 1993). Subsequent investigations have elucidated the molecular architecture, substrate selectivity, regulatory networks, and phylogenetic diversification of these membrane transporters (Sun et al. 2024; Maurel et al. 2015; Handa et al. 2022; Li et al. 2024). Current genomic studies have revealed a huge diversity of aquaporins in the plant kingdom, with the number of members ranging from 19 in *Selaginella moellendorffii* to 121 in *Brassica napus* (Rabeh et al. 2024; Anderberg et al. 2012; Yuan et al. 2017). These isoforms have been broadly categorized into five subfamilies: Plasma Membrane Intrinsic Proteins (PIPs), Tonoplast Intrinsic Proteins (TIPs), Nodulin 26-like Intrinsic Proteins (NIPs), Small and Basic Intrinsic Proteins (SIPs), and Uncharacterized X Intrinsic Proteins (XIPs). Among these, PIPs, TIPs, and NIPs represent evolutionarily conserved, functionally characterized groups ubiquitous in vascular plants. In contrast, SIPs—the smallest subfamily with limited water conductance—and XIPs exhibit restricted phylogenetic distribution, being notably absent in some higher plant species such as the monocots or the Brassicaceae (Maurel et al. 2015; Bezerra-Netoa et al. 2019).

Although recent reviews have extensively documented the structural diversity and evolution, subcellular localization, and physiological roles of aquaporins in plant development and stress adaptation (Sun et al. 2024; Maurel et al. 2015; Handa et al. 2022; Li et al. 2024; Wang et al. 2020; Tang et al. 2023; Rabeh et al. 2024; Bezerra-Netoa et al. 2019), critical knowledge gaps persist regarding their multi-layered regulation (Kapilan et al. 2018). This review synthesizes the most recent findings on spatiotemporal regulation of AQP gene expression, and stress-responsive protein modifications. It is worth noting that we focused solely on collecting examples for which direct evidence of the regulatory relationship exists (Table 1). By elucidating how plants orchestrate these regulatory networks to optimize water/nutrient flux, we provide novel insights into the evolutionary conservation and functional plasticity of aquaporin-mediated membrane transport systems.

**Table 1:** Summary of well-established regulators of AQPs in plants

Species	Regulators	Targeted AQPs	Regulatory action	Inductive signals	Assays	Refs
*Malus domestica*	MsDREB6.2	MdPIP1;3, Mdγ-TIP	Transcriptional activation	Drought	EMSA	(Liao et al. 2017)
*Fragaria* × *ananassa cv. Benihope*	RdreB1BI	FvPIP2;1 like 1	Transcriptional activation	Drought	Y1H	(Gu et al. 2017)
*Arabidopsis thaliana*	AtTG	AtTIP1;1, AtTIP2;3, AtPIP2;2	Transcriptional activation	Drought	EMSA, TAA	(Zhu et al. 2014)
*Pyrus betulaefolia*	PbERF3	PbPIP1;4	Transcriptional activation	Drought	Y1H, EMSA, DLA	(Zhang et al. 2024a)
*Arabidopsis thaliana*	AtPLATZ4	AtPIP2;8	Transcriptional repression	Drought	Y1H, ChIP-qPCR, DLA, EMSA	(Liu et al. 2023)
*Citrus sinensis*	CsMYB96	CsPIP1;1, CsPIP2;4	Transcriptional repression	Water loss	EMSA, DLA	(Zhang et al. 2022a)
*Citrus sinensis*	CsWRKY4	CsNIP5;1	Transcriptional activation	Water loss	Y1H, EMSA, DLA	(Zhang et al. 2022b)
*Citrus sinensis*	CsWRKY28	CsNIP5;1	Transcriptional repression	Water loss	Y1H, EMSA, DLA	(Zhang et al. 2022b)
*Phyllostachys edulis*	PeMYB99	PeTIP4-3	Transcriptional activation	Drought, salt	Y1H, EMSA, TAA, DLA	(Zhu et al. 2024)
*Tamarix hispida*	ThMYB8	ThTIP	Transcriptional activation	Salt	Y1H, TAA, ChIP-qPCR	(Liu et al. 2021)
*Tamarix hispida*	ThNAC12	ThPIP2;5	Transcriptional activation	Salt	Y1H, DLA, ChIP-PCR	(Wang et al. 2021)
*Musa acuminata*	MaERF1/39, MabZIP53, MaMYB22	MaPIP1;1	Transcriptional activation	Drought, salt, cold	Y1H, DLA	(Xu et al. 2021)
*Arabidopsis thaliana*	AtARR1, AtARR12	AtNIP1;1, AtNIP6;1	Transcriptional activation	Arsenite stress	ChIP–qPCR, EMSA, DLA	(Zhang et al. 2024b)
*Brassica napus*	BnaA9.WRKY47	BnaNIP5;1	Transcriptional activation	Boron deficiency	Y1H, EMSA, TAA	(Feng et al. 2019)
*Arabidopsis thaliana*	AtABI3	AtTIP3;1, AtTIP3;2	Transcriptional activation	Seed maturation	EMSA, DLA	(Mao and Sun 2015)
*Rosa hybrida*	RhNAC100	RhPIP1;1, RhPIP2;1	Transcriptional repression	Petal cell expansion	EMSA, ChIP-PCR	(Pei et al. 2013)
*Gossypium hirsutum*	GhACE1	GhPIP2;7	Transcriptional activation	Fiber elongation	EMSA, DLA ChIP-qPCR	(Lu et al. 2022a)
*Oryza sativa*	OsCPK17	OsPIP2;1, OsPIP2;6	Phosphorylation	Cold	Phosphoproteomic, in vitro phosphorylation	(Almadanim et al. 2017)
*Gentiana scabra*	GsCPK16	GsPIP2;2	Phosphorylation	Temperature and light stimuli	In vitro phosphorylation, *in cell* kinase assay	(Nemoto et al. 2022)
*Arabidopsis thaliana*	AtCPK34	AtNIP4;1, AtNIP4;2	Phosphorylation	Pollen development	in vitro phosphorylation, MS	(Di Giorgio et al. 2016)
*Arabidopsis thaliana*	AtCPK28	AtPIP2;7	Phosphorylation	*Phytophthora capsici* infection	In vitro and in vivo phosphorylation, MS	(Zhu et al. 2025)
*Arabidopsis thaliana*	AtOST1	AtPIP2;1-	Phosphorylation	ABA	In vitro phosphorylation	(Grondin et al. 2015)
*Arabidopsis thaliana*	AtOST1, AtBAK1	AtPIP2;1	Phosphorylation	ABA, flg22	In vitro phosphorylation	(Rodrigues et al. 2017)
*Arabidopsis thaliana*	AtSnRK2.4	AtPIP2;1	Phosphorylation	ABA, root hydraulics	In vitro phosphorylation	(Shahzad et al. 2023)
*Arabidopsis thaliana*	AtSIRK1	AtPIP2;1 etc	Phosphorylation	Sucrose, elicitor peptide 7	Phosphoproteomics	(Wu et al. 2019; Wu et al. 2013; Wang et al. 2022)
*Sorghum bicolor*	SbAT1	SbPIP2;1	Inhibited phosphorylation	Alkaline stress	MS	(Zhang et al. 2023b)
*Oryza sativa*	OsGS3	OsPIP2;1	Inhibited phosphorylation	Alkaline stress	In vivo phosphorylation	(Zhang et al. 2023b)
*Arabidopsis thaliana*	AtUBC32-AtRma1	AtPIP2;1	Ubiquitination	Drought	In vitro ubiquitination, MS	(Chen et al. 2021)
*Oryza sativa*	OsUBC45-DGS1	OsPIP2;1	Ubiquitination	*Magnaporthe orzae* infection	In vitro and in vivo ubiquitination	(Wang et al. 2023; Wang et al. 2024)
*Arabidopsis thaliana*	AtRma1H1	AtPIP2;1	Ubiquitination	Drought	In vivo ubiquitination	(Lee et al. 2009)
*Oryza sativa*	OsRINGzf1	OsPIP2;1	Ubiquitination	Drought	In vitro ubiquitination	(Chen et al. 2022)
*Oryza sativa*	OsHIR1	OsTIP4;1	Ubiquitination	Arsenic, cadmium	In vitro ubiquitination	(Lim et al. 2014)

*ABA* Abscisic acid, *ChIP* Chromatin immunoprecipitation, *DLA* Dual luciferase reporter assay, *EMSA* Electrophoretic mobility shift assay, *MS* mass spectrometry, *TAA* transient activation assay, *Y1H* Yeast one-hybrid assay

## Transcriptional regulation of *AQPs* in plants

**Fig. 1:**
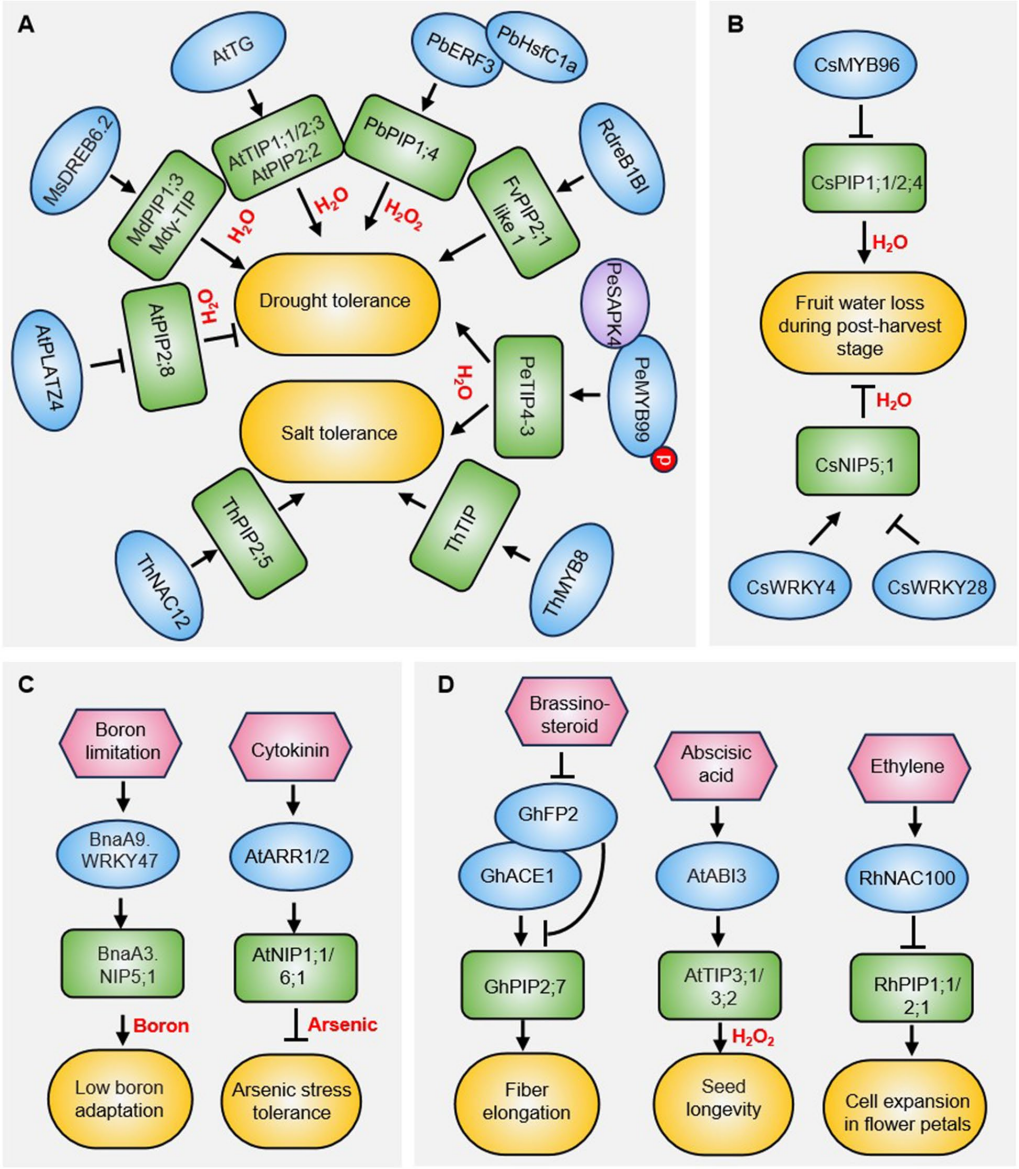
Transcriptional regulatory network of *AQPs* established in plants. **A** Transcriptional regulation of *AQPs* under drought and salinity stress conditions. **B** Transcriptional regulation of *AQPs* during postharvest fruit dehydration. **C** Transcriptional regulation of *AQPs* under heavy metal stress. **D** Transcriptional regulation of *AQPs* during phytohormone regulated plant growth and development. Transport substrates for designated AQPs are highlighted in red. Positive regulatory relationships are indicated by solid black arrows, while negative regulatory interactions are denoted by perpendicular black bars. Protein–protein interactions are represented by spatial co-localization of the corresponding elements. Phosphorylation events are denoted by red circles containing "p" symbols

Expression profiling has revealed that the transcription of plant *AQP* genes undergoes significant changes during stress responses and developmental processes. Several upstream transcription factors governing *AQPs* expression have been identified and characterized (Fig. 1, Table 1), providing insights into the transcriptional mechanisms underlying *AQP* gene regulation in plants.

### Transcriptional regulation of *AQPs* in response to abiotic stresses

The AP2/ERF (APETALA2/ethylene-responsive element binding factors) transcription factors (TFs) are a large group of TFs widely present in plants. Among them, the dehydration response element binding protein (DREB) is the most extensively studied group involved in abiotic stress (Feng et al. 2020). In apple (*Malus domestica*), overexpression of *MsDREB6.2* results in decreased stomatal aperture, density and increased root hydraulic conductance, thereby enhancing drought tolerance in transgenic plants (Liao et al. 2017). Under drought conditions, the expression of *MdPIP1;3* and *Mdγ-TIP* was dramatically increased in the *35S:MsDREB6.2* plants but decreased in the *35S:MsDREB6.2SRDX* plants. Further experiments demonstrated that *MdPIP1;3* and *Mdγ-TIP* are direct target genes of MsDREB6.2 and participate in the MsDREB6.2-mediated increase in root hydraulic conductance upon drought stress (Liao et al. 2017). Similarly, in strawberry (*Fragaria* × *ananassa* cv. Benihope), RdreB1BI can bind to the promoter of *FvPIP2;1-like 1*, and activate its expression to modulate plant drought stress response (Gu et al. 2017). In Arabidopsis (*Arabidopsis thaliana*), the AP2/ERF family TF TRANSLUCENT GREEN (TG) directly binds to the promoters of three aquaporin genes (*AtTIP1;1*, *AtTIP2;3*, and *AtPIP2;2*), to activate their expression and thereby controlling water balance (Zhu et al. 2014). In pear (*Pyrus betulaefolia*), PbERF3 interacts with PbHsfC1a and coordinately binds to the *PbPIP1;4* promoter to activate it, thus stimulating the H₂O₂ signaling pathway to confer drought tolerance (Zhang et al. 2024a). These findings suggest that AP2/ERF TFs play a crucial role in plant drought tolerance by transcriptional activation of *AQP* genes.

The PLATZ4 (Plant A/T-rich protein and zinc-binding protein 4) TF from Arabidopsis mediates transcriptional repression of plasma membrane aquaporin *AtPIP2;8*, thereby reducing drought tolerance by inhibiting stomatal closure (Liu et al. 2023). In postharvest fruit, water loss leads to rind discoloration, reduced weight/firmness, and accelerated senescence. In citrus (*Citrus sinensis*), expression profiling indicated that *CsPIP1;1* and *CsPIP2;4* had high expression that was representative of other aquaporins, and they were down-regulated in the peel of post-harvest citrus fruit (Zhang et al. 2022a). Transient overexpression of *CsPIP2;4* significantly enhanced water loss in citrus fruit. Through in vivo and in vitro analyses, CsMYB96 was demonstrated to directly suppress *CsPIP1;1* and *CsPIP2;4* expression as a defense mechanism against dehydration during postharvest stage (Zhang et al. 2022a). Conversely, the overexpression of *CsNIP5;1* alleviated water loss in both stored citrus fruit and citrus callus (Zhang et al. 2022b). Further studies identified CsWRKY4 and CsWRKY28 as direct transcriptional regulators of *CsNIP5;1*, functioning as opposing modulators —CsWRKY4 activates while CsWRKY28 represses it (Zhang et al. 2022b).

In moso bamboo (*Phyllostachys edulis*), *PeTIP4-3* is predominantly expressed in shoots, particularly within the vascular bundle sheath cells. Overexpression of *PeTIP4-3* enhances drought and salt tolerance in transgenic plants (Zhu et al. 2024). A MYB-family TF PeMYB99 binds directly to the *PeTIP4-3* promoter, activating its expression and further amplifying tolerance to drought and salt stress. Additionally, the kinase PeSAPK4 interacts with and phosphorylates PeMYB99, synergistically boosting *PeTIP4-3* transcription (Zhu et al. 2024). A parallel regulatory mechanism is observed in *Tamarix hispida*, where ThMYB8 improves salt stress tolerance by binding to the MBSI motif (CAACTG) in the promoter of *ThTIP* and activating its expression (Liu et al. 2021). Similarly, an NAC (no apical meristem [NAM], Arabidopsis transcription activation factor [ATAF], and cup-shaped cotyledon [CUC])-domain TF ThNAC12 directly binds to the NAC recognition sequence in the *ThPIP2;5* promoter, upregulating *ThPIP2;5* expression to enhance salt tolerance via ROS scavenging modulation (Wang et al. 2021). In banana (*Musa acuminata*), overexpression of *MaPIP1;1* confers enhanced tolerance to drought, salinity, and low-temperature stress in transgenic lines (Xu et al. 2021). Further studies identified MaERF1/39, MabZIP53, and MaMYB22 as upstream TFs that bind to *MaPIP1;1* promoter, regulating its expression and influencing stress tolerance in banana (Xu et al. 2021).

### Transcriptional regulation of *AQPs* in response to metal ion toxicity

Plant hormone cytokinin regulates diverse physiological processes including growth, development, and stress responses (Cortleven et al. 2019). It was revealed that AtARR1 and AtARR12 (type-B Arabidopsis response regulators) acted as negative regulators to participate in cytokinin signaling-mediated AS(III) response in *Arabidopsis* (Zhang et al. 2024b). This regulatory axe acts upstream of two NIP-encoding genes, *AtNIP1;1* and *AtNIP6;1,* which are critical for arsenic uptake and transport. By suppressing *AtNIP1;1*/*6;1* expression, AtARR1/12 balance arsenic accumulation, minimizing toxicity while maintaining essential nutrient homeostasis (Zhang et al. 2024b). In *Brassica napus*, the transcription factor BnaA9.WRKY47 binds strongly to the W-box motif in promoters of boron transport-associated gene *BnaNIP5;1*, directly regulating its expression to optimize boron content (Feng et al. 2019).

### Transcriptional regulation of *AQPs* during plant growth and development

Aquaporins are known to regulate water transport during plant tissue expansion in several key developmental processes, such as seed maturation and fiber elongation (Mao and Sun 2015; Pei et al. 2013; Lu et al. 2022a). In Arabidopsis, *AtTIP3;1* and *AtTIP3;2* are specifically expressed during seed maturation and contribute to seed longevity. The B3 TF AtABI3 (Abscisic acid insensitive 3) directly binds to the RY motif in the promoters of *AtTIP3* genes to activate their expression, thereby enhancing seed desiccation tolerance and longevity (Mao and Sun 2015). In rose (*Rosa hybrida*), the NAC-domain TF RhNAC100 binds to the promoters of *RhPIP1;1* and *RhPIP2;1* to repress them, which in turn promotes ethylene-mediated cell expansion in flower petals (Pei et al. 2013). In cotton (*Gossypium hirsutum*), the bHLH/HLH TF GhACE1 (Activator for cell elongation 1) directly activates the expression of *GhPIP2;7* by binding to its promoter, thereby regulating fiber elongation. GhFP2 (Fiber-related protein 2) interacts with GhACE1, and suppresses the transcriptional activation of GhACE1 toward *GhPIP2;7*. Interestingly, BR signaling-activated GhBZR1 (Brassinazole-resistant 1) could suppress the transcription of GhFP2 directly, preventing GhFP2 from forming heterodimers with GhACE1, thus releasing its inhibition to GhACE1 (Lu et al. 2022a).

## Post-translational regulation of AQPs

Post-translational modification (PTM) is a biochemical process in which specific amino acid residues of target proteins are covalently attached to functional chemical groups. In plants, AQPs were found to be modified by phosphorylation, ubiquitination, methylation and N-terminal acetylation. The modified-AQPs can be stabilized, degraded, activated, or inactivated depending on the specific time and location when the functional group has been added. To date, only the regulatory mechanisms underlying AQP phosphorylation and ubiquitination have been partially elucidated; therefore, we focused on these two modifications.

### Regulation of AQPs by phosphorylation

Kinase-catalyzed phosphorylation of serine or threonine residues in proteins plays a critical role in signal transduction during plant growth and environmental adaptation (Zhang et al. 2023a). In diverse plant species, conserved phosphorylation sites have been identified within loops A–E and the N- and C-terminal regions of aquaporins (Pietro et al. 2013). Numerous studies have established that phosphorylation is a significant regulatory mechanism of AQPs as it influences their activity, localization or trafficking, substrate selectivity, and interaction with other integral cellular components under various stress conditions (Prak et al. 2008; Prado et al. 2013, 2019; Qing et al. 2016; Qiu et al. 2020; Chen et al. 2021; Lu et al. 2022b). For instance, in wheat (*Triticum aestivum*), phosphorylation of TaPIP2;10 at Ser-280 enhances CO₂ uptake into cells, boosting photosynthetic efficiency and grain yield. Conversely, under pathogen or insect attack, apoplastic H₂O₂ triggers phosphorylation of TaPIP2;10 at Ser-121. This modification enables H₂O₂ transport into the cytoplasm, where it amplifies host defense responses and restricts further invasion (Lu et al. 2022b).

**Fig. 2:**
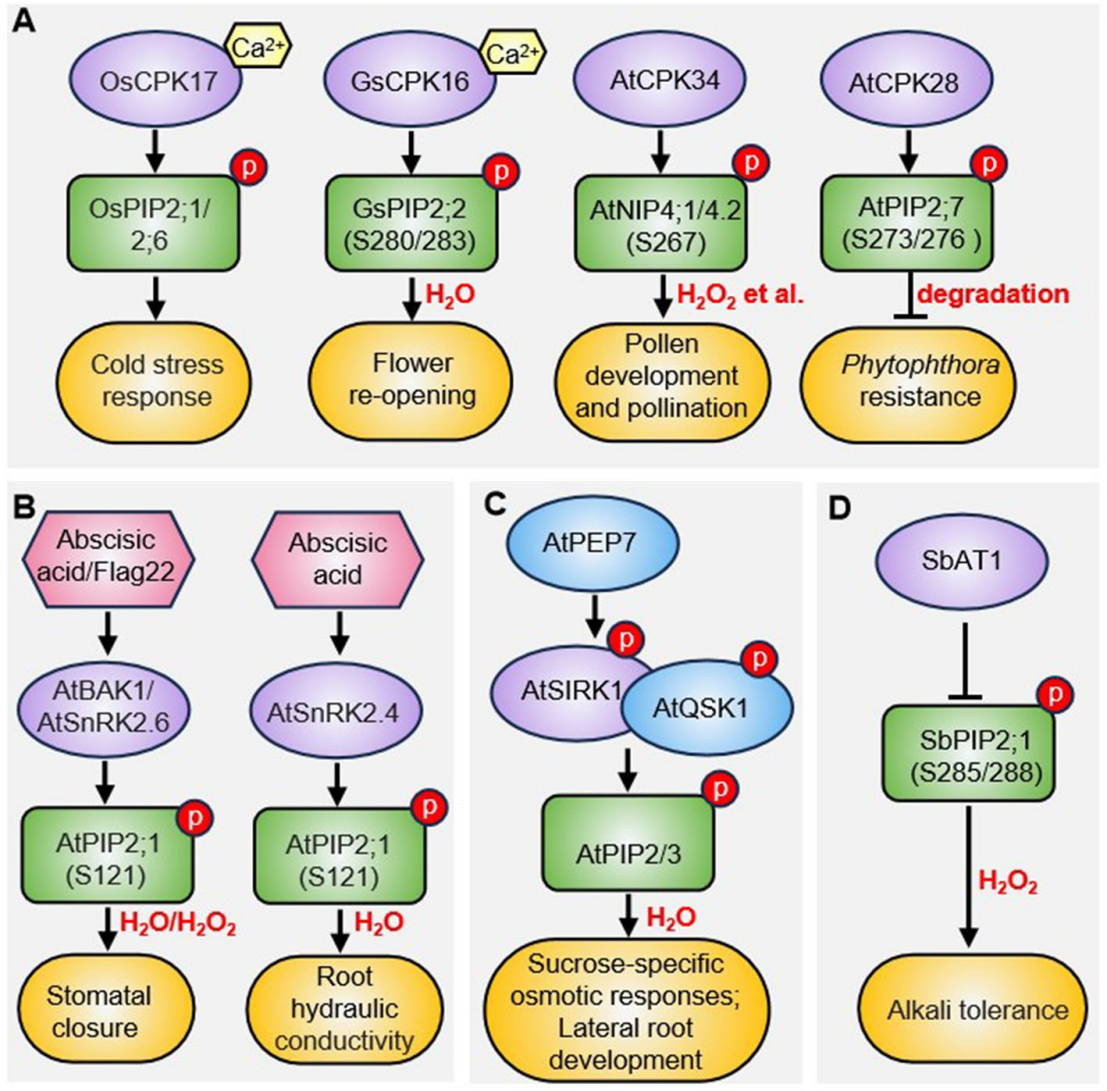
Simplified schematic of kinase-mediated AQP phosphorylation in plant development and stress responses. **A** CDPK-mediated phosphorylation of AQPs during plant stress responses and developmental processes. **B** ABA- or flg22-activated kinases modulate AQP phosphorylation to regulate stomatal closure and root hydraulic conductivity. **C** PEP7-SIRK1-QSK1 signaling axis for AQP phosphorylation during sucrose-specific osmotic responses and lateral root development. **D** AT1-mediated repression of AQP phosphorylation reduces plant alkali stress tolerance. Phosphorylation events are denoted by red circles containing "p" symbols. Kinase activation is indicated by solid black arrows, while inhibitory effects are marked by black blocked lines. Protein interactions are represented by spatial co-localization of the corresponding elements

Protein kinase A (PKA) was the first kinase identified to phosphorylate AQPs and enhance their water channel activity in plants (Temmei et al. 2005; Van Wilder et al. 2008; Maurel et al. 1995). Recent studies have revealed several additional kinases that directly regulate AQP phosphorylation (Fig. 2). Calcium-dependent protein kinases (CPKs) can sense and be activated by calcium (Ca^2^⁺), a ubiquitous second messenger, thus widely involved in stress response regulation in plants (Valmonte et al. 2014). For example, in rice (*Oryza sativa*), OsCPK17 phosphorylates OsPIP2;1/2;6 in a Ca^2^⁺-dependent manner, enabling cold stress adaptation (Almadanim et al. 2017). Similarly, in gentian (*Gentiana scabra*), GsCPK16 phosphorylates GsPIP2;2 in response to cytosolic Ca^2^⁺ fluctuations triggered by temperature and light stimuli, facilitating flower re-opening (Nemoto et al. 2022). In Arabidopsis, the pollen-specific aquaporins AtNIP4;1/4;2, which function as water and small neutral solute channels, are phosphorylated at Ser-267 in their C-terminal regions by the pollen-specific kinase AtCPK34, modulating their water permeability (Di Giorgio et al. 2016). Additionally, AtCPK28 interacts with and phosphorylates AtPIP2;7 at Ser-273/Ser-276, promoting its degradation under normal conditions (Zhu et al. 2025). During pathogen infection, however, AtCPK28 dissociates from AtPIP2;7 and becomes destabilized, leading to AtPIP2;7 accumulation to bolster defense responses (Zhu et al. 2025).

Stomatal movements in response to environmental stimuli are critically controlled by plant water status (Ding et al. 2024). Abscisic acid (ABA) induces stomatal closure by doubling the osmotic water permeability of guard cell protoplasts and triggering reactive oxygen species (ROS) accumulation in guard cells—both effects are eliminated in *atpip2;1* mutant (Grondin et al. 2015). AtOST1 (Open stomata 1), a kinase central to ABA signaling in guard cells, phosphorylates AtPIP2;1 at Ser-121. This phosphorylation enhances AtPIP2;1-mediated water transport. Expression of a phosphomimic AtPIP2;1 (Ser121Asp)—but not the phosphodeficient variant (Ser121Ala)—in *atpip2;1* plant constitutively elevated guard cell protoplast water permeability, bypassed ABA-dependent activation, and restored ABA-induced stomatal closure (Grondin et al. 2015). Similarly, the pathogen-associated molecular pattern (PAMP) flg22 requires AtPIP2;1 to induce stomatal closure. The flg22 increases guard cell protoplast water permeability via AtPIP2;1 activation, a process dependent on both AtBAK1 (Brassinosteroid insensitive 1-associated receptor kinase 1) and AtOST1, which co-phosphorylate AtPIP2;1 at Ser-121. flg22-triggered H₂O₂ accumulation and stomatal closure were rescued in *atpip2;1* guard cell by the Ser121Asp variant but not Ser121Ala (Rodrigues et al. 2017). These findings establish plasma membrane aquaporins as signaling hubs in ABA- and flg22-mediated stomatal closure (Grondin et al. 2015; Rodrigues et al. 2017). Additionally, the Class 2 Sucrose Non-Fermenting Protein Kinase AtSnRK2.4 interacts with and phosphorylates AtPIP2;1 at Ser-121, enhancing its water transport activity to fine-tune root hydraulic conductivity (Shahzad et al. 2023).

Another key regulator of AQP phosphorylation is the plasma membrane-localized sucrose-induced receptor kinase 1 (AtSIRK1) and its co-receptor AtQSK1 (Qian Shou kinase 1) (Wu et al. 2019, 2013; Wang et al. 2022). AtSIRK1 undergoes autophosphorylation to initiate downstream signaling and trans-phosphorylates AtQSK1, which subsequently interacts with and phosphorylates AQPs (Wu et al. 2019, 2013). Recent studies identified elicitor peptide 7 (AtPEP7), a member of the plant elicitor peptide (PEP) family, as the specific ligand of AtSIRK1 (Wang et al. 2022). Upon sucrose treatment, AtPEP7 binds to the extracellular domain of AtSIRK1, triggering in vivo AQP phosphorylation and enhancing water influx into protoplasts. Impaired water influx in *pep7* mutants consequently delays lateral root development. Notably, the *sirk1* loss-of-function mutant fails to respond to exogenous PEP7, exhibiting no activation of kinase activity, AQP phosphorylation, water influx, or lateral root development (Wang et al. 2022). These findings establish the PEP7/SIRK1/QSK1 complex as a central signaling module mediating sucrose-regulated water transport and lateral root morphogenesis in Arabidopsis (Wang et al. 2022).

Interestingly, SbAT1 (Alkaline Tolerance 1) was recently cloned as a major locus governing plant alkaline tolerance in sorghum (*Sorghum bicolor*) (Zhang et al. 2023b). SbAT1 encodes an atypical G protein γ subunit. IP-MS analysis revealed that SbAT1 interacts with multiple aquaporins, notably SbPIP2;1/2;2 and SbPIP1;3/1;4, to negatively regulating their phosphorylation and subsequent hydrogen peroxide (H₂O₂) distribution (Zhang et al. 2023b). More importantly, SbAT1 shares 55.56% protein sequence identity with rice OsGS3 (Grain size 3) and 82.30% identity with maize ZmGS3, indicating evolutionary conservation of its regulatory mechanism. Notably, OsGS3 similarly interacts with OsPIP2;1 and its homolog OsPIP2;2 to reduce their phosphorylation levels, thereby negatively regulating rice alkaline tolerance (Zhang et al. 2023b). Although the precise mechanism by which AT1 suppresses AQP phosphorylation remains unclear, this study underscores the critical role of AQPs and their phosphorylation status in plant alkaline stress adaptation.

### Regulation of AQPs by ubiquitination-mediated degradation

**Fig. 3:**
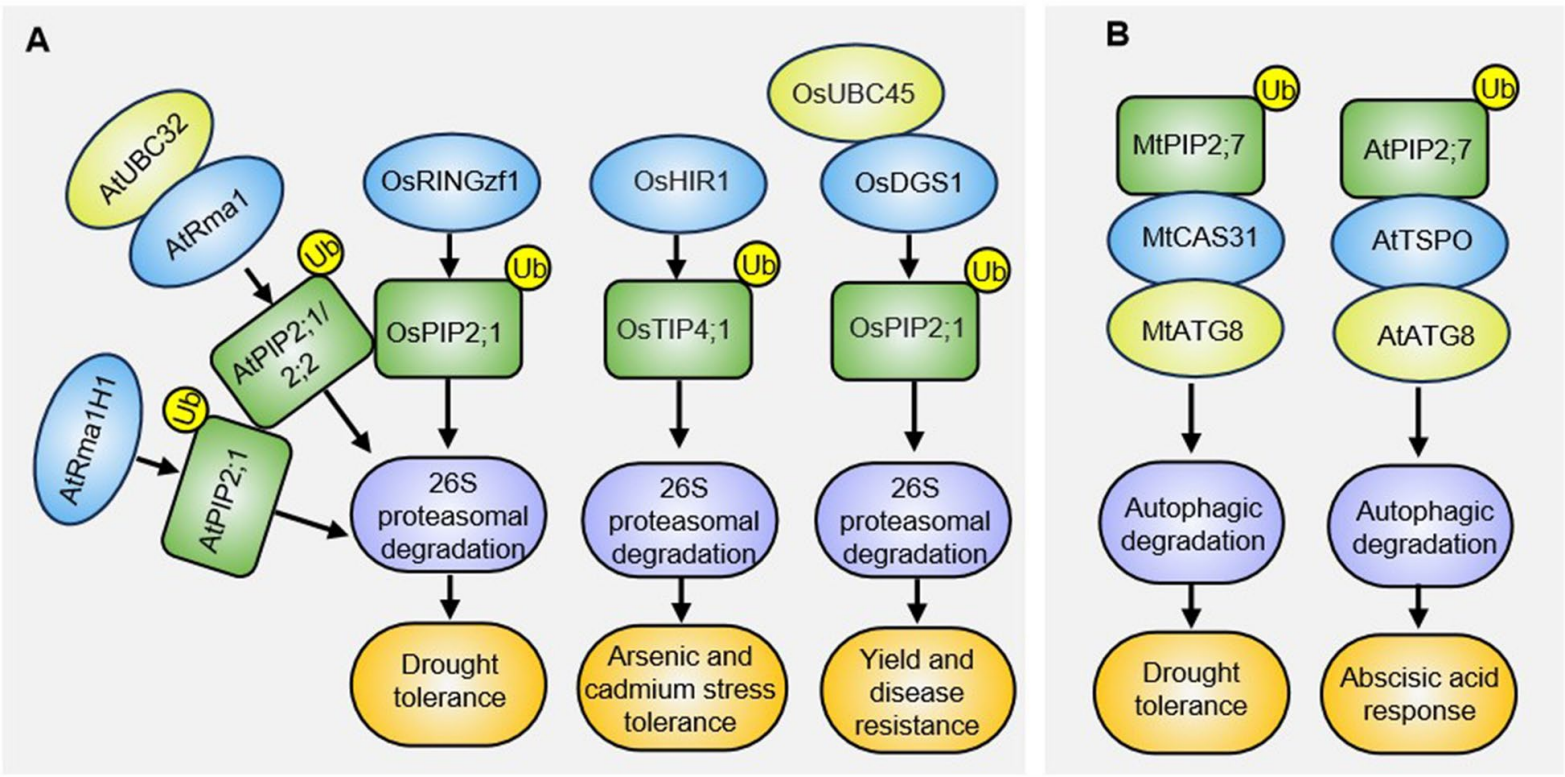
Simplified schematic of ubiquitination-mediated AQP degradation in plant development and stress responses. **A** Identified E3 ligases or E2-E3 pairs mediating AQP ubiquitination for 26S proteasomal degradation. **B** Selective autophagy-regulated vacuolar trafficking and degradation of AQPs. Ubiquitination events are denoted by black circles containing "Ub" symbols. Protein interactions are represented by spatial co-localization of the corresponding elements

Ubiquitination is one type of highly conserved post-translational modifications. It involves the covalent attachment of ubiquitin molecules to lysine residues of diverse protein substrates. This enzymatic process is mediated through a three-enzyme cascade: ubiquitin-activating enzyme (E1), ubiquitin-conjugating enzyme (E2), and ubiquitin ligase (E3) (Suranjika et al. 2024). The best characterized ubiquitination pathway is the ubiquitin-26S proteasome system, which plays essential roles during AQPs homeostasis (Fig. 3A).

The endoplasmic reticulum-associated protein degradation (ERAD) is one of the ubiquitin proteasome systems that degrades misfolded or unfolded proteins accumulated in ER to alleviate ER stress (Liu and Howell 2016). In Arabidopsis, AtUBC32 act as a critical ubiquitin-conjugating enzyme (E2) in the ERAD pathway. It partners with the RING-type E3 ligase AtRma1 to ubiquitinate the aquaporin AtPIP2;1 at lysine 276, marking it for degradation via the 26S proteasome (Chen et al. 2021). This regulatory mechanism reduces AtPIP2;1 abundance, enhancing plant drought tolerance by modulating water transport efficiency. Interestingly, the C-terminal (Ser-280 and Ser-283) phosphorylated form of AtPIP2;1 is the active form, which exhibits a faster degradation rate than the nonphosphorylated form, indicating the interplay between phosphorylation and ubiquitination in determining AtPIP2;1 stabilization (Chen et al. 2021). Similarly, in rice (*Oryza sativa*), the ubiquitin-conjugating enzyme OsUBC45 participates in the ERAD pathway to promote broad-spectrum disease resistance and improve yield. OsUBC45 facilitates the degradation of the aquaporin OsPIP2;1, which negatively regulates pathogen-associated molecular pattern (PAMP)-triggered immunity by suppressing defense responses (Wang et al. 2023). Recent studies reveal that DGS1 (Decreased grain size 1), a RING-type E3 ligase, forms an E2–E3 pair with OsUBC45 to mediate the ubiquitin-dependent degradation of OsPIP2;1, thereby enhancing rice immunity (Wang et al. 2024).

In Arabidopsis, drought stress-induced AtRma1H1, a RING membrane-anchor E3 ubiquitin ligase, interacts with and ubiquitinates AtPIP2;1 in vitro and in vivo. The ubiquitination leads to destabilization of AtPIP2;1 and this process was inhibited by 26S proteasome inhibitor MG132 (Lee et al. 2009). The AtRma1H1-mediated ubiquitination also inhibited the trafficking of AtPIP2;1 from the ER to the plasma membrane (Lee et al. 2009). Similar regulatory mechanisms also exist in rice (Chen et al. 2022). *OsRINGzf1* is a drought-responsive gene encoding a RING zinc finger protein possessing E3 ubiquitin ligase activity. In *OsRINGzf1*-overexpressing rice plants, the protein levels of OsPIP2;1 were significantly reduced. Notably, this degradation was effectively blocked by the proteasome inhibitor MG132, demonstrating the crucial role of the ubiquitin–proteasome pathway in regulating OsPIP2;1 stability (Chen et al. 2022). Since OsPIP2;1 is essential for transmembrane water transport, OsRINGzf1-mediated degradation of this aquaporin reduces cellular water permeability, thereby enhancing water conservation under drought stress (Chen et al. 2022). In addition, rice heavy metal responsive E3 ligase OsHIR1 (Heavy metal-induced RING E3 ligase 1) physically interacts with the tonoplast aquaporin OsTIP4;1 at the plasma membrane. Through 26S proteasome-dependent destabilization of OsTIP4;1, OsHIR1 modulates arsenic and cadmium uptake, revealing an important protein degradation mechanism for environmental adaptation in rice (Lim et al. 2014).

Besides the 26S proteasome pathway, autophagy is another important protein quality control mechanism. It employs specialized vesicles to encapsulate and deliver cytoplasmic material (including molecules, protein aggregates, or even organelles) to the vacuole for breakdown. In this process, ubiquitination frequently serves as a specific recruitment signal to ensure precise cargo selection (Marshall and Vierstra 2018). Emerging evidence highlights the autophagic degradation of AQPs as a critical mechanism for plant stress adaptation (Fig. 3B) (Hachez et al. 2014; Li et al. 2019). In Arabidopsis, AtPIP2;7 was shown to interact in the ER and Golgi, with a membrane protein, named TSPO for tryptophan-rich sensory protein/translocator and serving as a multistress regulator. The complex was then directed towards vacuolar degradation via the autophagosome pathway through direct binding of AtTSPO with AtATG8s (Autophagy-related gene 8) (Hachez et al. 2014). This process is stimulated by the drought-induced hormone abscisic acid, which reduces root hydraulic conductivity to minimize water loss and improve drought tolerance (Hachez et al. 2014). In *Medicago truncatula*, dehydrin MtCAS31 (Cold acclimation-specific 31) interacts with MtATG8a and positively regulates drought response through its integral involvement in the autophagic degradation pathway (Li et al. 2019). MtPIP2;7 is identified as a cargo protein of MtCAS31, which facilitates the autophagic degradation of MtPIP2;7 (Li et al. 2019). These findings collectively establish autophagy as a vital quality control system for AQPs regulation in plants. Notably, while ubiquitination participates in cargo recognition, the E3 ligases mediating AQPs ubiquitination in autophagic contexts remain unidentified, representing a key target for future research.

## Conclusion

Aquaporins (AQPs) are essential membrane channels, facilitating water and small solute transport to regulate plant growth and environment adaptation. Significant progress has been made in understanding the multiple facets of plant AQPs, including their structural diversity, functions, and regulation. Their expression is transcriptionally controlled by stress-/developmental responsive TFs such as AP2/ERF, MYB, NAC, and WRKY, which activate or repress *AQP* genes under abiotic stresses (e.g., drought, salinity) or during specific developmental stages (e.g., seed maturation) (Fig. 1, Table 1). Based on these findings, water stress resilience in crops has been achieved by manipulating the expression of specific aquaporin isoforms in plant tissues functional under water-deficit conditions (Ahmed et al. 2021; Singh et al. 2024). Transcriptional regulation usually operates synergistically with epigenetic mechanisms like DNA methylation, histone modifications (e.g., acetylation, methylation), and non-coding RNAs (Zhang and Zhu 2025). These epigenetic processes dynamically modulate chromatin accessibility, either enabling or obstructing TF binding to DNA—for instance, DNA methylation often silences genes by compacting chromatin, while histone acetylation promotes an open chromatin state to enhance transcriptional activation (Zhang and Zhu 2025). In humans, MicroRNAs have been identified as important endogenous modulators of AQP expression involved in several diseases (Gomes et al. 2018), but similar information is still lacking in plants. Future studies should identify epigenetic regulators that fine-tune *AQP* expression, offering insights into chromatin-level control of AQPs during plant growth and stress responses.

In-depth mass spectrometry analyses have revealed that plant AQPs carry numerous post-translational modifications, mainly including phosphorylation and ubiquitination. Phosphorylation by kinases (e.g., CPKs, SnRK2s) (Fig. 2, Table 1) enhances AQPs water permeability or stress signaling, whereas ubiquitination by E3 ligases (e.g., AtRma1, OsHIR1) (Fig. 3, Table 1) targets AQPs for proteasomal/autophagic degradation, optimizing water-use efficiency and heavy metal detoxification. In future, the molecular mechanisms and regulatory complexity of PTMs on AQPs still require further investigation. This includes studying the interplay between phosphorylation and ubiquitination during AQPs regulation, as well as the spatiotemporal dynamics of reversible modifications (e.g., phosphorylation/dephosphorylation, ubiquitination/deubiquitylation) and their impact on AQP’s actions such as transport activity, localization or trafficking. Besides phosphorylation and ubiquitination, other PTMs such as methylation and N-terminal acetylation were also found to occur in plant AQPs (Santoni et al. 2006; Vera-Estrella et al. 2004). For example, methylation of AtPIP2;1 does not interfere with its intrinsic water permeability but affects its trafficking (Santoni et al. 2006). Meanwhile, N-acetylation of ice plant (*Mesembryanthemum crystallinum*) McTIP1;2 seems to act on its redistribution to endosomal compartments (Vera-Estrella et al. 2004). However, the detailed mechanisms leading to these modifications and their roles are yet unknown. Addressing these questions will deepen our understanding of how PTMs fine-tune AQPs function under fluctuating environmental conditions. A deeper understanding of AQP’s functions and regulatory mechanisms could provide new strategies for optimizing plant growth and development, enhancing stress resistance, and improving crop yields.

## Data Availability

All data generated or analyzed during this study are included in this published article.

## Abbreviations

ABA: Abscisic acid
ABI3: Abscisic acid insensitive 3
ACE1: Activator for cell elongation 1
AP2/ERF: APETALA2/ethylene-responsive element binding factors
AQPs: Aquaporins
ARR: Arabidopsis response regulator
AT1: Alkaline tolerance 1
ATG8: Autophagy-related gene 8
BAK1: Brassinosteroid insensitive 1-associated receptor kinase 1
BZR1: Brassinazole-resistant 1
CAS31: Cold acclimation-specific 31
CPK: Calcium-dependent protein kinase
DGS1: Decreased grain size 1
DREB: Dehydration response element binding protein
ERAD: Endoplasmic reticulum-associated protein degradation
FP2: Fiber-related protein 2
GS3: Grain size 3
HIR1: Heavy metal-induced ring E3 ligase 1
NAC: No apical meristem [NAM], Arabidopsis transcription activation factor [ATAF], and cup-shaped cotyledon [CUC]
NIPs: Nodulin 26-like intrinsic proteins
OST1: Open stomata 1
PAMP: Pathogen-associated molecular pattern
PEP: Elicitor peptide
PIPs: Plasma membrane intrinsic proteins
PKA: Protein kinase A
PLATZ4: Plant A/T-rich protein and zinc-binding protein 4
PTMs: Post-translational modifications
QSK1: Qian Shou kinase 1
ROS: Reactive oxygen species
SIPs: Small and basic intrinsic proteins
SIRK1: Sucrose-induced receptor kinase 1
TFs: Transcription factors
TG: Translucent green
TIPs: Tonoplast intrinsic proteins
TSPO: Tryptophan-rich sensory protein/translocator
XIPs: Uncharacterized X intrinsic proteins
